# Prediction of prognosis in glioblastoma with radiomics features extracted by synthetic MRI images using cycle-consistent GAN

**DOI:** 10.1007/s13246-024-01443-8

**Published:** 2024-06-17

**Authors:** Hisanori Yoshimura, Daisuke Kawahara, Akito Saito, Shuichi Ozawa, Yasushi Nagata

**Affiliations:** 1 Department of Radiation Oncology, Graduate School of Biomedical and Health Sciences, Hiroshima University, Hiroshima, 734-8551 Japan; 2 Department of Radiology, National Hospital Organization Kure Medical Center, Hiroshima, Japan; 3 Hiroshima High-Precision Radiotherapy Cancer Center, Hiroshima, 732-0057 Japan

**Keywords:** Neural network, GAN, Cycle-GAN, Radiomics, Machine learning

## Abstract

To propose a style transfer model for multi-contrast magnetic resonance imaging (MRI) images with a cycle-consistent generative adversarial network (CycleGAN) and evaluate the image quality and prognosis prediction performance for glioblastoma (GBM) patients from the extracted radiomics features. Style transfer models of T1 weighted MRI image (T1w) to T2 weighted MRI image (T2w) and T2w to T1w with CycleGAN were constructed using the BraTS dataset. The style transfer model was validated with the Cancer Genome Atlas Glioblastoma Multiforme (TCGA-GBM) dataset. Moreover, imaging features were extracted from real and synthesized images. These features were transformed to rad-scores by the least absolute shrinkage and selection operator (LASSO)-Cox regression. The prognosis performance was estimated by the Kaplan–Meier method. For the accuracy of the image quality of the real and synthesized MRI images, the MI, RMSE, PSNR, and SSIM were 0.991 ± 2.10 $\times {10}^{-4}$, 2.79 ± 0.16, 40.16 ± 0.38, and 0.995 ± 2.11 $\times {10}^{-4}$, for T2w, and .992 ± 2.63 $\times {10}^{-4}$, 2.49 ± 6.89 $\times {10}^{-2}$, 40.51 ± 0.22, and 0.993 ± 3.40 $\times {10}^{-4}$ for T1w, respectively. The survival time had a significant difference between good and poor prognosis groups for both real and synthesized T2w (p < 0.05). However, the survival time had no significant difference between good and poor prognosis groups for both real and synthesized T1w. On the other hand, there was no significant difference between the real and synthesized T2w in both good and poor prognoses. The results of T1w were similar in the point that there was no significant difference between the real and synthesized T1w. It was found that the synthesized image could be used for prognosis prediction. The proposed prognostic model using CycleGAN could reduce the cost and time of image scanning, leading to a promotion to build the patient’s outcome prediction with multi-contrast images.

## Introduction

Primary brain tumors are subdivided into different categories [1]. Among them, glioblastoma (GBM) is known to be one of the most lethal and refractory primary brain tumors. The GBM has a median survival of just 15 month after diagnosis. Only past exposure to ionizing radiation and certain genetic syndromes are recognized as risk factors for GBM [2]. Standard therapy consists of surgical resection to the extent, which is safely feasible, followed by radiotherapy [3]. In a clinical trial using fractionated radiotherapy followed by adjuvant temozolomide; the median survival time is still less than two years [4]. The endpoint for the evaluation of treatment is primarily prolongation of overall survival (OS) [5].

There are several methods for predicting OS. Conventionally, factors affecting the prognosis include high tumor grade, multiple lesions, age, sex, Karnofsky performance score, the extent of resection, treatment plan, and several biomarkers [6–8]. Regarding the risk of GBM, ionizing radiation is one of the few known risk factors that reliably indicates an increased risk of glioma development [8]. In addition, radiation-induced GBM is typically seen years after therapeutic radiation, which is indicated for another tumor or condition [5]. Including vinyl chloride, pesticides, smoking, petroleum refining, and synthetic rubber manufacturing have been loosely associated with the development of GBM.

In addition to clinical factors, MRI images have been used for prognosis prediction [9–17]. Image-based computational models play an increasingly important role in precise diagnosis and treatment guidance in neuro-oncology [18]. Recently, quantitative imaging features derived from medical imaging were of added value in predicting outcome parameters in oncology in what is called Radiomics. We have been working with the University of Minnesota to develop a prognosis prediction system [19]. Local control of brain metastasis was predicted, and the proposed method was more accurate than visual evaluation. For the GBM patients, there are several reports on GBM Radiomics using multi-modality MRI images [20–23]. Pak et al. proposed a prognosis prediction model obtaining imaging features from the dynamic contrast enhanced MRI image [24]. In addition, Shim et al. proposed a deep learning-based distant recurrence prediction model using T1-weighted MRI image (T1w), contrast-enhanced T1-weighted MRI images (CE-T1w), fluid attenuated inversion recovery MRI image (FLAIR), and dynamic susceptibility contrast-enhanced MRI images [21]. On the other hand, problems with Radiomics include the time consuming to scan for the multi-contrast MRI image scan, the dependence of some features on scan acquisition and image reconstruction parameters, and the fact that the best model varies with sample size [25, 26].

The correction methods for variation of the imaging features have been proposed with normalization and combat techniques [27]. On the other hand, images with artifacts from MRI imaging are excluded because they could not correct and affect the accuracy of the prognosis prediction [28, 29]. These problems are expected to be solved by new deep learning techniques such as style transfer and improved normalization, which will make MRI more robust [30, 31]. In addition, the style transfer could synthesize missing images [29]. Several algorithms for style transfer have been proposed [31, 32]. Recent studies proposed style transfer techniques using a generative adversarial network (GAN) [33]: pix2pix [34] and cycle-consistent generative adversarial network (CycleGAN) [40]. The style transfer models of MRI images were proposed for the medical images [36–39]. These studies developed a highly accurate method for synthesizing medical images, which contributes to improving radiation diagnosis performance, such as detecting lesions using synthetized images.

Recently, there has been a movement to use style transfer techniques such as GAN to synthesize images and use the synthesized images for Radiomics [40–42]. Comparing previous study with ours, de Farias et al. and Chen et al. both implemented CT super-resolution in GAN and performed radiomics analysis on the synthesized CT images [40, 41]. In our study, we perform T1w and T2w transformations, so although the task of image transfer is similar, we consider them to be different studies in terms of synthesizing different contrasts. In addition, Tixier et al. generated images using MRI images, however used it to harmonize and normalize images across multiple centers [42]. The purpose of this study differs from that of Tixier et al. study, as it synthesized MRI images of different contrasts to predict OS. Islam et al. aimed to complement missing MRI images by synthesizing them [29]. In contrast, this study predicts OS using only the imaging features of the synthesized image. In contrast, we predicted the OS using the real and synthesized MRI images. From these comparisons, the novelty of this study is that it predicts OS even with synthesized images alone. A paper on prognosis prediction using Radiomics with synthesized images has been reported [29]. They synthesized the missing modality and proposed the survival rate prediction. However, there has been not reported image quality and contribution as a predictor of the synthesized MRI images by comparing with the real MRI images. Also, Kaplan–Meier survival analysis has a benefit to divide survival risk by considering time in many small intervals [43].

The current study proposes a CycleGAN-based T1w and T2 weighted MRI image (T2w) style transfer model for GBM patients.

Moreover, we predicted the OS in preoperative images of GBM patients between the real and synthesized MRI images with Kaplan–Meier survival analysis. A feasibility of the style transfer model was evaluated by comparing with the real and synthesized MRI images in the point of image quality and the survival prediction performance.

## Material and methods

**Fig. 1:**
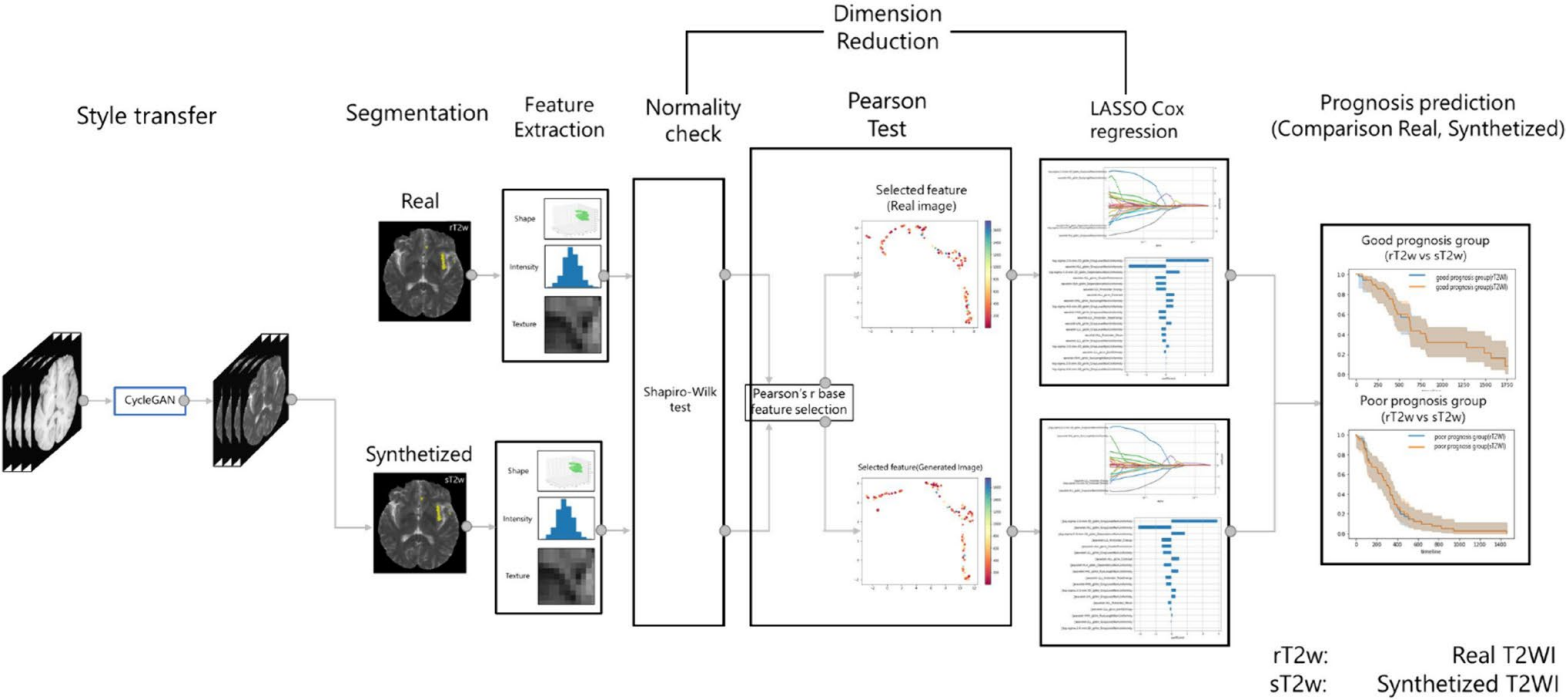
Overview of the process of Radiomics-based prognosis prediction models using style transfer. The prognosis prediction model constructed consists of the following processes: Style transfer, Segmentation, Feature Extraction, Dimension Reduction, Prognosis prediction. Dimension Reduction is further composed of parts Normality check, Pearson Test, and LASSO Cox regression. The upper part of the figure shows Real image, and the lower part shows the flow of the synthesized image

Figure 1 shows the overall workflow from style transfer to prognosis prediction using Radiomics analysis in the current study. The detail of the dataset is described in ‘Dataset’section. The style transfer is described in ‘Style transfer’ section. The evaluation of image quality is described in ‘Assessment of style transfer with radiomics analysis’section. In addition, the assessment of style transfer with Radiomics analysis is described in ‘Statistical analysis’ section.

### Dataset

#### Training dataset for style transfer

**Fig. 2:**
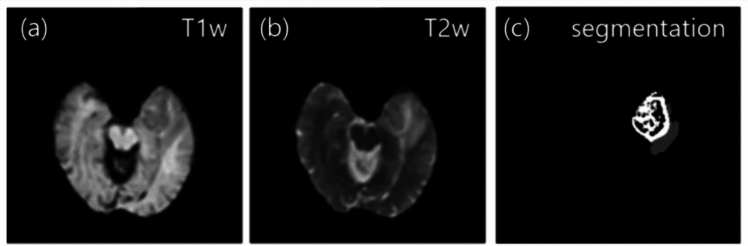
Training dataset for style transfer of (**a**) T1w, (**b**) T2w, and (**c**) tumor segmentation

We used the Brain Tumor Segmentation Challenge (BraTS) dataset for the style transfer model building. The BraTS provided a large, annotated dataset of low-grade gliomas and high-grade GBMs to encourage the development of modern methods of tumor segmentation. T1w, CE-T1w, T2w, FLAIR, and tumor segmentation were provided for each case. The current study used T1w and T2w from the BraTS 2017 dataset [44]. In this paper, 150 cases from the BraTS 2017 dataset were used. Figure 2 shows an example of the images used in this paper.

#### Test dataset for style transfer/radiomics dataset

**Fig. 3:**
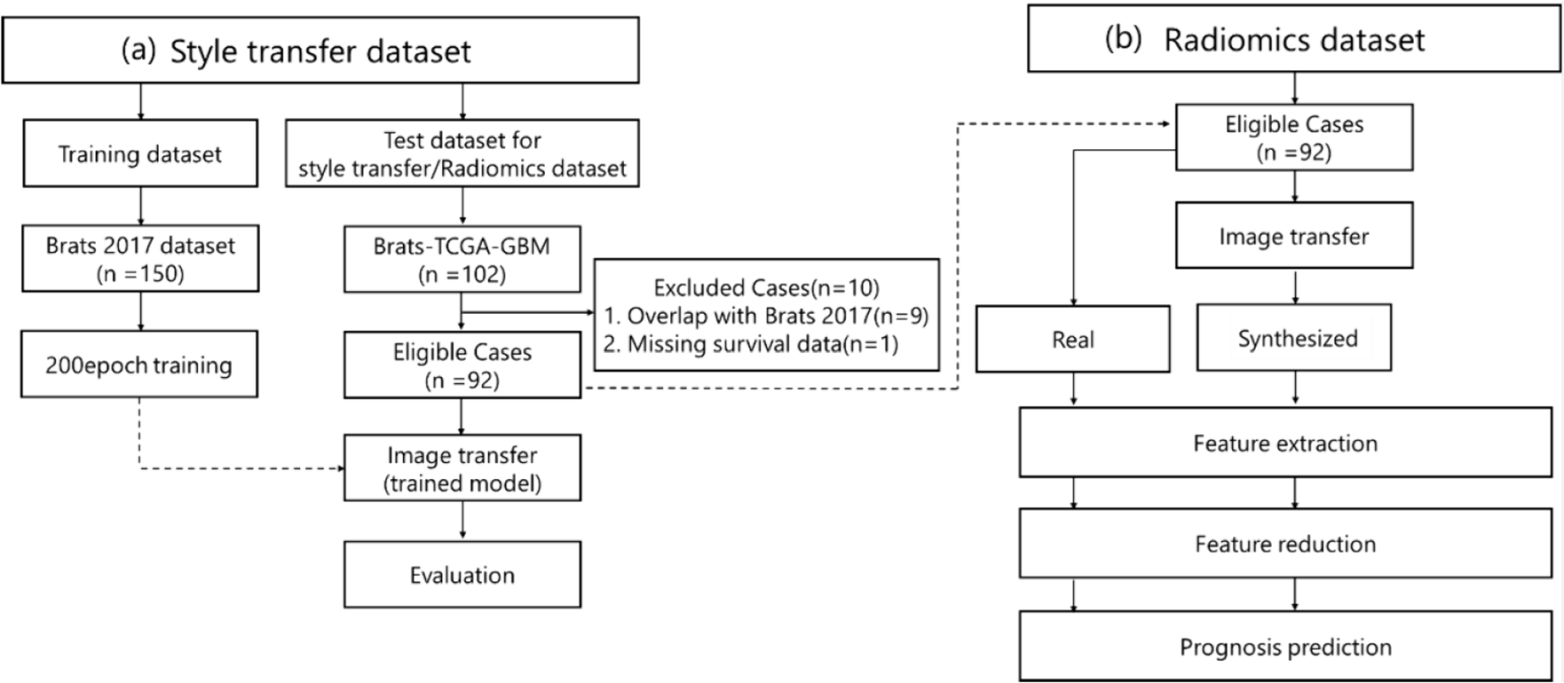
Flowchart of patient exclusion for (**a**) style transfer dataset and (**b**) Radiomics dataset using BraTS-TCGA-GBM

In this paper, we use the third-party data of The Cancer Genome Atlas (TCGA) GBM collection (TCGA-GBM) [45]. We used the test dataset called “Segmentation Labels and Radiomic Features for Pre-operative Scans of TCGA-GBM collection (BraTS-TCGA-GBM)” [46] as a test dataset for style transfer and Radiomics analysis. The test dataset of 102 cases contains the same kind of multi contrast MRI images and segmentation as the training dataset. Figure 3(a) shows the workflow for excluding cases from the style transfer dataset. The training dataset used is BraTS 2017 above. Figure 3(a) also shows the process of evaluating the synthesized image. The exclusion of cases was done as follows. First, the test dataset includes some BraTS 2017; 9 overlapping cases were excluded. Next, 1 case with missing survival time data was excluded. Finally, the remaining 92 cases were used as the test dataset, which was used for building the prognosis prediction model with Radiomics analysis. As with the training dataset, T1w and T2w were used. Style transfer and synthesized image evaluation will be presented in subsequent sections. Figure 3(b) shows the feature extraction process to the prognosis prediction from real and synthesized images using the test dataset. Details of each process are discussed in the subsequent sections.

### Style transfer

**Fig. 4:**
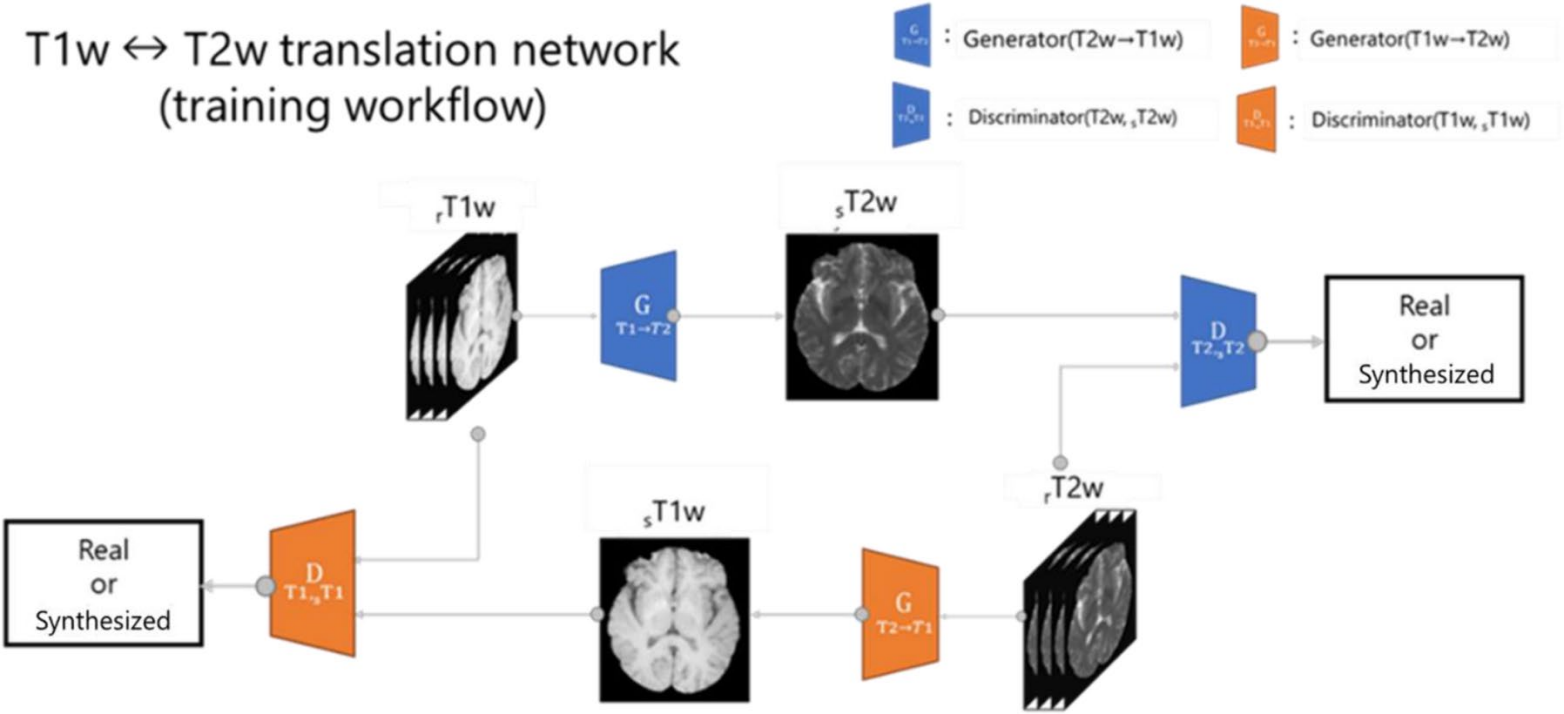
T1w to T2w and T2w to T1w translation networks using CycleGAN. rT1w and rT2w are real image sT2w and sT1w are synthesized image. G_T1→T2_ and G_T2→T1_ are the generator. The generator produces a synthesized image by transforming the contrast of the image. D_T1, sT1_ and D_T2, sT2_ are the discriminator. The discriminator distinguishes between real image and synthesized images

Figure 4 shows the network that converts T1w and T2w using CycleGAN. To distinguish between the real image and the synthesized image, the real T2w is denoted as “rT2w” and the synthesized T2w as “sT2w”. Simultaneously, the style transfer model of the T2w to T1w image was constructed as the input of the T2w. The real T1w is denoted as “rT1w”, and the synthesized T1w is denoted as “sT1w”.

**Fig. 5:**
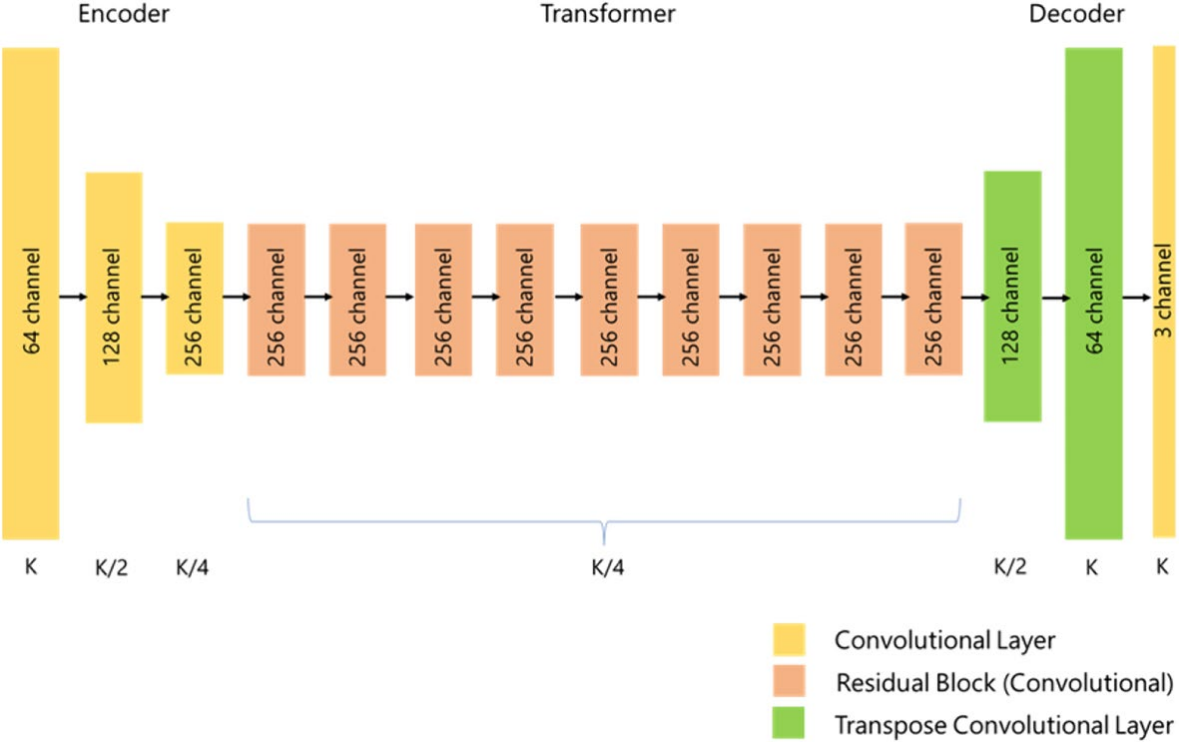
An architecture for the CycleGAN generator. The representation size shrinks in the encoder phase, stays constant in the transformer phase, and expands again in the decoder phase. The representation size that each layer outputs is listed below it, in terms of the input image size, K. On each layer is listed the number of channels. Each layer is followed by an instance normalization and Rectified Linear Unit (ReLU) activation function

**Fig. 6:**
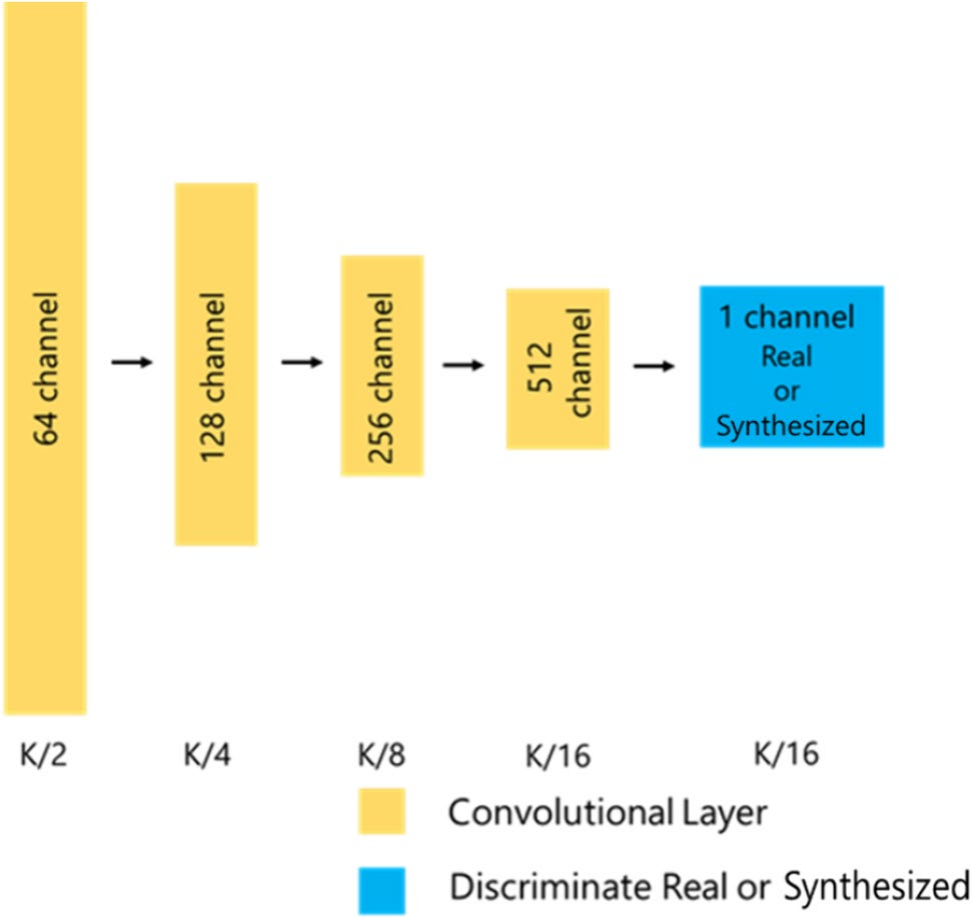
An architecture for the CycleGAN discriminator. It calls PatchGAN to discriminate real and synthesized images for each region cut out by the patch size. The representation size from each layer is listed below each layer in terms of the input image size, K. On each layer is listed the number of channels

The CycleGAN has two different networks, generator (G) and discriminator (D), which are the basic structure of GAN, shown in Figs. 5 and 6, respectively. The structure of *G* and *D* is different from conventional GAN. The CycleGAN uses a special *G* based on U-Net [47], used in several segmentation studies [48–50]. It can synthesize highly accurate images by adding the ResNet [51] structure to the U-Net. In this paper, 9-layer ResNet was used. In addition, CycleGAN uses a special D called PatchGAN [34], which was also used in pix2pix, to stabilize the learning process. PatchGAN discriminates real and synthesized images for each region cut out by the patch size.

More detailed definitions of GAN and CycleGAN are given below. First, GAN is a method that trains two different networks, *G* and *D*, alternately to obtain highly accurate images. The learning of GAN is described as

1 $$\underset{G}{\text{min}}\,\underset{D}{\text{max}}V\left(D,G\right)= {\mathbb{E}}_{x\sim {p}_{\text{data}}\left(x\right)}\left[\text{log}\left(D\left(x\right)\right)\right]+{\mathbb{E}}_{z\sim {p}_{z}\left(z\right)}\left[\text{log}\left(\left(1-D(G\left(z\right)\right)\right)\right]$$

where GAN is characterized by playing a mini-max game of this function.

Next, learning a GAN is described as

2 $${L}_{\text{GAN}}\left(G, {D}_{Y}, X, Y\right)={\mathbb{E}}_{y\sim {p}_{\text{data}}\left(y\right)}\left[\text{log}\left({D}_{Y}\left(y\right)\right)\right]+{\mathbb{E}}_{x\sim {p}_{\text{data}}\left(x\right)}\left[\text{log}\left(\left(1-{D}_{Y}(G\left(x\right)\right)\right)\right]$$

where CycleGAN learns *G*, which synthesized a highly accurate virtual image *G*(*X*), using adversarial loss.

However, for learning GAN, adversarial loss alone is not binding. Therefore, CycleGAN introduces the concept of cycle constancy. The cycle constancy is described as

3 $${L}_{\text{cyc}}\left(G,F\right)={\mathbb{E}}_{x\sim {p}_{\text{data}}\left(x\right)}\left[{\Vert F\left(G\left(x\right)\right)-x\Vert }_{1}\right]+{\mathbb{E}}_{x\sim {p}_{\text{data}}\left(y\right)}\left[{\Vert G\left(F\left(y\right)\right)-y\Vert }_{1}\right]$$

where *F* is a function that recovers the distribution *X* by inverse transformation, as in *F*(*G*(*x*)).

Here, the learning of CycleGAN including cycle constancy is described as

4 $$L\left(G,F, {D}_{X}, {D}_{Y}\right)={L}_{\text{GAN}}\left(G,{D}_{Y}, X, Y \right)+ {L}_{\text{GAN}}\left(F, {D}_{X}, Y,X \right)+ {\lambda L}_{\text{cyc}}\left(G,F\right)$$

Finally, learning a CycleGAN is described as

5 $${G}^{*},{F}^{*}=\text{arg}\,\underset{G,\mathit{ }F}{\text{min}}\underset{{D}_{x},{D}_{Y}}{\text{max}}L\left(G,F, {D}_{X}, {D}_{Y}\right)$$

where CycleGAN is characterized by playing a mini-max game of $L\left(G,F, {D}_{X}, {D}_{Y}\right)$, just like learning GAN.

The proposed model was implemented using the Ubuntu operating system (18.04) with PyTorch packages (V1.4.0, CUDA 11.2, Python 3.7.12). Moreover, the CycleGAN used the following implementation [52]. Hyper parameter was calculated with the default settings. A model to convert between T1w and T2w was trained with identical hyperparameters and instance normalization. A minibatch of 2D MRI images was randomly selected from the training set at each iteration. We calculated at 200 epochs, although most studies usually stop the calculation at 50 epochs [35, 53, 54]. Moreover, we also used methods “Mini-batch processing”, “Normalization and penalization”, and “Scheduling of learning rate” to prevent mode collapse. These processes were used to run on a 16-GB NVIDIA Tesla P100 GPU.

### Evaluation of image quality

The following formula was used to create a difference image, and the synthesized image was visually evaluated:

$$\text{difference}= {\left(r\left(i,j\right)-t(i,j)\right)}^{2},$$

where $r(i,j)$ is the value of the pixel $(i,j)$ in the real image, $t(i,j)$ is the value of pixel $(i,j)$ in the synthesized image.

The difference image was normalized by the following formula:

$$Y= \frac{\left({x}_{i}-\text{min}\left({x}_{i}\right)\right)}{\left(\text{max}\left({x}_{i}\right)-\text{min}\left({x}_{i}\right)\right)},$$

where ${x}_{i}$ is the value of pixel, and $\text{min}\left({x}_{i}\right)$ and $\text{max}\left({x}_{i}\right)$ are the minimum and maximum value of pixel.

The prediction accuracy of the model for synthesized images was evaluated using the following four metrics: mutual information (MI), root mean square error (RMSE), signal-to-noise ratio (PSNR), and structural similarity index (SSIM). These metrics are defined as follows:

The $\text{MI}(X, Y)$ is used as a cross-modality similarity measure. It is calculated as

6 $$\text{MI}(X, Y) =\sum_{x\in X}\sum_{y\in Y}{P}_{X,Y}\left(x,y\right)\text{log}\frac{{P}_{X,Y}(x,y)}{{P}_{X}(x){P}_{Y}(y)}$$

where ${P}_{X,Y}$ is the joint probability mass function of $X$ and $Y$, and ${P}_{X}$ and ${P}_{Y}$ are the marginal probability mass functions of $X$ and $Y$, respectively.

The RMSE is defined as

7 $$\text{RMSE}= \sqrt{\frac{1}{{n}_{x}{n}_{y}}\sum_{i, j}^{{n}_{x}{n}_{y}}{\left(\frac{r(i,j)- t(i,j)}{r(i,j)}\right)}^{2}}$$

where $r(i,j)$ is the value of the pixel $(i,j)$ in the real image, $t(i,j)$ is the value of pixel $(i,j)$ in the synthesized image, and ${n}_{x}{n}_{y}$ is the total number of pixels.

The PSNR is calculated as

8 $$\text{PSNR}=20 \bullet {\text{log}}_{10}\frac{\text{MAX}}{\sqrt{\text{MSE}}}$$

where MSE was mean square error. The MSE is calculated as the square of RMSE.

Here, the SSIM between two images, $\overrightarrow{x}$ and $\overrightarrow{y}$, can be computed as in Wang et al. [55]:

9 $$\text{SSIM}\left(\text{x},\text{ y}\right)= \frac{(2{\mu }_{x}{\mu }_{y} + {c}_{1})(2{\sigma }_{xy}+ {c}_{2})}{\left({{\mu }_{x}}^{2}+ {{\mu }_{y}}^{2}+ {c}_{1}\right)\left({{\sigma }_{x}}^{2}+ {{\sigma }_{y}}^{2}+ {c}_{2}\right)}$$

10 $${C}_{1}= {({k}_{1}Q)}^{2}, {k}_{1}=0.01$$

11 $${C}_{2}= {({k}_{2}Q)}^{2}, {k}_{2}=0.03$$

where ${C}_{1}$ and ${C}_{2}$ are constants used to prevent a zero denominator and to maintain the stability of the formula. $Q$ is the maximum value for the real and synthesized images. The values of ${k}_{1}$ and ${k}_{2}$ are typically obtained from Qi et al. [56]. ${\sigma }_{x}$ is an estimate in the discrete form:

12 $${\sigma }_{x}=\sqrt{\frac{1}{N-1}\sum_{i=1}^{N}{({x}_{i}-{\mu }_{x})}^{2}}$$

The covariance between $\overrightarrow{x}$ and $\overrightarrow{y}$ is defined as ${\sigma }_{xy}$. It is defined as

13 $${\sigma }_{xy}= \frac{1}{N-1}\sum_{i=1}^{N}\left({x}_{i}-{\mu }_{x}\right)\left({y}_{i}-{\mu }_{y}\right)$$

where ${\mu }_{x}$ is the mean intensity and can be expressed as

14 $${\mu }_{x}= \frac{1}{\text{N}}\sum_{i=1}^{\text{N}}{x}_{i}$$

These metrics calculated each case got the average and standard deviation (SD). SD is defined as

15 $$\text{SD}= \sqrt{\frac{1}{n}\sum_{i=1}^{n}{({x}_{i}-\overline{x })}^{2}}$$

where $\overline{x }$ is average and *n* is the number of data.

### Assessment of style transfer with radiomics analysis

#### Feature extraction

The target image was used as the stacked 2D image of rT2w, sT2w, rT1w, and sT1w to extract 3D imaging features. A segmentation data was used from the BraTS-TCGA-GBM. We extracted imaging features using PyRadiomics [57].

These features were extracted from the tumor regions on rT2w, rT1w and synthesized MRIs sT2w, sT1w, respectively. These features include shapes, first-order, second-order (gray level co-occurrence matrix (GLCM), gray level size zone matrix (GLSZM), gray level run length matrix (GLRLM), and gray level dependence matrix (GLDM) features), and higher-order (Laplacian of Gaussian (LoG) filter and wavelet transform).

#### Dimension reduction

The normality was confirmed by the Shapiro–Wilk test [58] for these features extracted from real and synthesized images, respectively. Then, referring to the analysis by delta-Radiomics, Pearson’s *r* was calculated for imaging features of real and synthesized images [59]. The Pearson’s *r* is calculated as

16 $${\rho }_{X,Y}=\frac{{\sigma }_{xy}}{{\sigma }_{X}{\sigma }_{Y}}$$

where ${\sigma }_{X}$ and ${\sigma }_{Y}$ are the standard deviation of $X$ and $Y$, respectively. These features with Pearson’s *r* greater than 0.9 were removed.

In this study, we used the least absolute shrinkage and selection operator (LASSO) regression with the Cox proportional hazards model (LASSO-Cox regression). The LASSO-Cox regression is a data analysis method that can shrink the coefficients of variables unrelated to survival time to zero [60]. These features with non-zero coefficients were selected based on tenfold cross-validation.

These selected features with real and synthesized images were converted to Radiomics score (rad-score) [61]. The rad-score transformation is defined as

17 $$\widehat{\beta }=\text{argmin}\left[{\ell}\left(\beta \right)\right],\text{ subject to }\sum \left|{\beta }_{j}\right|\le s$$

where $\widehat{\beta }$ is the obtained parameters, ${\ell}\left(\beta \right)$ is the log partial likelihood of the logistic regression model, *s* > 0 is a constant.

#### Prognosis prediction

Survival curves were estimated from the rad-score with real and synthesized images for each case. These survival curves were estimated by the Kaplan–Meier method [62]. The Kaplan–Meier method is defined as

18 $$\widehat{S}\left(t\right)= \prod_{i: {t}_{i}\le t}\left(1-\frac{{d}_{i}}{{n}_{i}}\right)$$

where ${t}_{i}$ is the time at which at least one event occurred, ${d}_{i}$ is the number of events (e.g., deaths) that occurred at time ${t}_{i}$, and ${n}_{i}$ is the number of individuals that have survived (i.e., events have not yet occurred or been censored) until time ${t}_{i}$. In addition, a log-rank (LR) test [63] was performed to determine if the hazard functions of the two samples are equal with the median of the rad-score as the cutoff. Let ${h}_{1}\left(t\right)$ and ${h}_{2}\left(t\right)$ be the two hazard functions, and define the null hypothesis as

19 $${\text{H}}_{0} : {h}_{1}\left(t\right)= {h}_{2}\left(t\right)$$

and the alternative hypothesis as

20 $${\text{H}}_{0} : {h}_{1}\left(t\right)\ne {h}_{2}\left(t\right)$$

To construct a test statistic, let ${r}_{1j}$ and ${r}_{2j}$ be the numbers of observations just before time ${t}_{j}$ in samples 1 and 2, with ${r}_{j}={r}_{1j}+{r}_{2j}$. Finally, let ${i}_{j}$ be a variable that takes the value 1 if the event at time ${t}_{j}$ occurs in sample 1, and 0 otherwise. Then, the LR test is given as

21 $${Z}_{\text{LR}}=\frac{{\sum }_{j=1}^{n}{i}_{j}-{\sum }_{j=1}^{n}\frac{{r}_{1j}}{{r}_{j}}}{\sqrt{{\sum }_{j=1}^{n}\frac{{r}_{1j}\bullet {r}_{j2}}{{{r}_{j}}^{2}}}}$$

where, ${Z}_{\text{LR}}$ can be approximated by a normal distribution. When ${z}_{\text{obs}}$ is the observed value of the test statistic, the null hypothesis can be rejected if $2P\left({Z}_{\text{LR}}\ge {z}_{\text{obs}}\right)\le 0.05$.

### Statistical analysis

All the statistical analyses were achieved with Python 3.7.12. The Shapiro–Wilk test was implemented using SciPy 1.7.1. Pearson’s r was implemented using the Numpy 1.19.5. Cox’s proportional hazard’s model with the LASSO penalty was implemented using the scikit-survival 0.17.1. The Kaplan–Meier method and LR test were implemented using lifelines 0.26.4.

## Results

### Style transfer

**Fig. 7:**
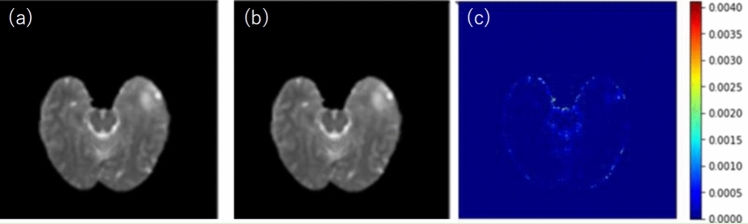
Example of a style transfer testing: (**a**) rT2w, (**b**) sT2w, and (**c**) difference image between rT2w and sT2w. The color bar of the difference image was set to the maximum value of the difference image

Figure 7 shows examples of rT2w, style transfer testing results for the sT2w using BraTS-TCGA-GBM dataset, and the difference image between rT2w and sT2w. The color bar of the difference image was set to the maximum value of the difference image. A more detailed numerical evaluation of the accuracy of sT2w is summarized in Table 1. The MI, RMSE, PSNR, and SSIM between rT2 and sT2 were 0.991 ± 2.10 $\times {10}^{-4}$, 2.79 ± 0.16, 40.16 ± 0.38, and 0.995 ± 2.11 $\times {10}^{-4}$, respectively.

**Table 1:** Average mutual information (MI), average root mean square error (RMSE), average peak signal-to-noise ratio (PSNR), and average structural similarity index measure (SSIM) computed from the synthesized T2w from the T1w

	MI	RMSE	PSNR	SSIM
Average SD	0.9912.10 $\times {10}^{-4}$	2.790.16	40.160.38	0.9952.11 $\times {10}^{-4}$

**Fig. 8:**
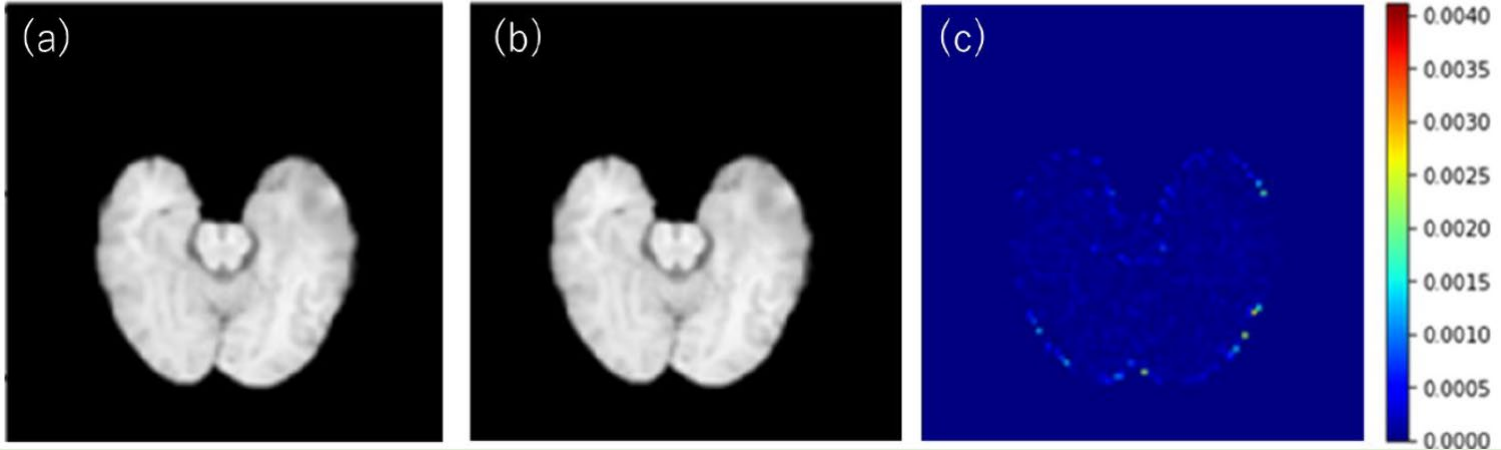
Example of a style transfer testing: (**a**) rT1w, (**b**) sT1w, and (**c**) difference image between rT1w and sT1w. The color bar of the difference image was set to the maximum value of the difference image

Figure 8 shows examples of rT1w, style transfer testing results for the sT1w using BraTS-TCGA-GBM dataset, and the difference image between rT1w and sT1w. The color bar of the difference image was set to the maximum value of the difference image. A more detailed numerical evaluation of the accuracy of sT1w is summarized in Table 2. The MI, RMSE, PSNR, and SSIM between rT1 and sT1 were 0.992 ± 2.63 $\times {10}^{-4}$, 2.49 ± 6.89 $\times {10}^{-2}$, 40.51 ± 0.22, and 0.993 ± 3.40 $\times {10}^{-4}$, respectively.

**Table 2:** Average mutual information (MI), average root mean square error (RMSE), average peak signal-to-noise ratio (PSNR), and average structural similarity index measure (SSIM) computed from the synthesized T1w from the T2w

	MI	RMSE	PSNR	SSIM
Average SD	0.9922.63 $\times {10}^{-4}$	2.4886.89 $\times {10}^{-2}$	40.510.22	0.9933.40 $\times {10}^{-4}$

### Prediction model with radiomics analysis

For the Radiomics analysis, imaging features were extracted from rT2w, sT2w, rT1w, and sT1w. 1720 features were extracted from the original image and the image with the LoG filter (σ = 2.0 to 5.0 [mm]), and 2752 features were extracted from the image with the wavelet transform. A total of 4528 features were extracted.

These imaging features had no significant difference from the results of the Shapiro–Wilk test (*p* ≥ 0.05). The Shapiro–Wilk test was performed once for each image feature.

**Fig. 9:**
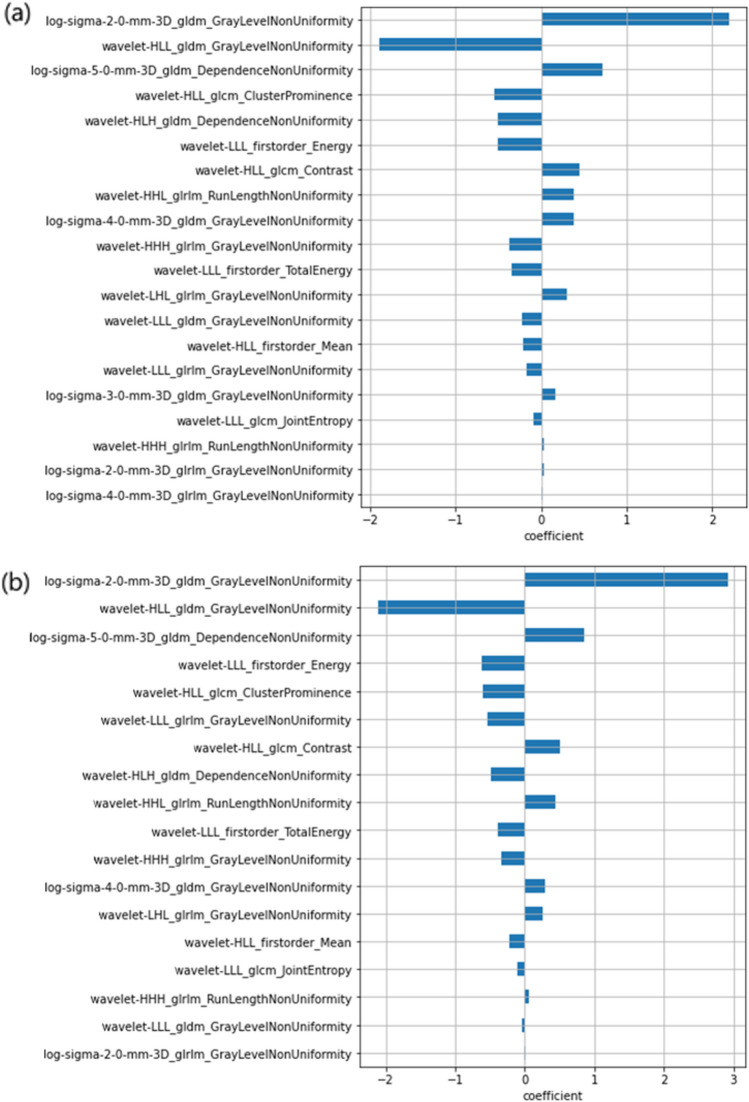
LASSO-Cox regression selected features (**a**) rT2w and (**b**) sT2w. The features selected in (**a**) and (**b**), respectively, have coefficients obtained by Lasso-cox regression. These coefficients are used to construct the Rad-score

For rT2w and sT2w, 64 features remained after eliminating these features with Pearson’s r greater than 0.9. The LASSO-Cox regression resulted in 20 features for rT2w and 18 features for sT2w. Figure 9 lists the selected features from the LASSO-Cox regression. The same 18 features for rT2w and sT2w were selected. These features included local variability and NonUniformity.

**Fig. 10:**
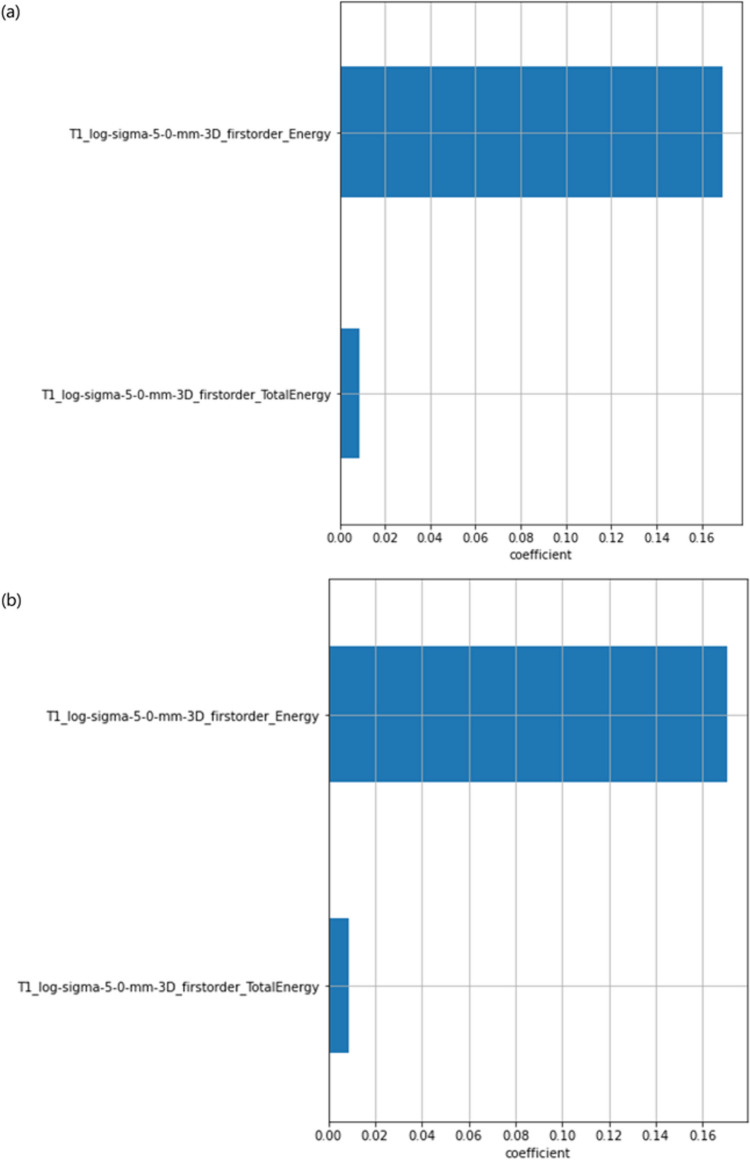
LASSO-Cox regression selected features (**a**) rT1w and (**b**) sT1w. The features selected in (**a**) and (**b**), respectively, have coefficients obtained by Lasso-cox regression. These coefficients are used to construct the Rad-score

For rT1w and sT1w images, 23 features remained after eliminating these features with Pearson’s r greater than 0.9. The LASSO-Cox regression resulted in 2 features for rT1w and 2 features for sT1w. Figure 10 lists these selected features from the LASSO-Cox regression. These same 2 features for rT1w and sT1w images were selected. The names of these features were log-sigma-5–0-mm-3D_firstorder_Energy and log-sigma-5–0-mm-3D_firstorder_TotalEnergy.

### Prognosis prediction

To evaluate the ability of the rad-score to prognosis prediction, patients were divided into two groups according to the median rad-score. Of the two groups, the group with the higher rad-score was designated as the poor prognosis group with a shorter survival time. The group with a lower rad-score was designated as the good prognosis group with a longer survival time.

**Fig. 11:**
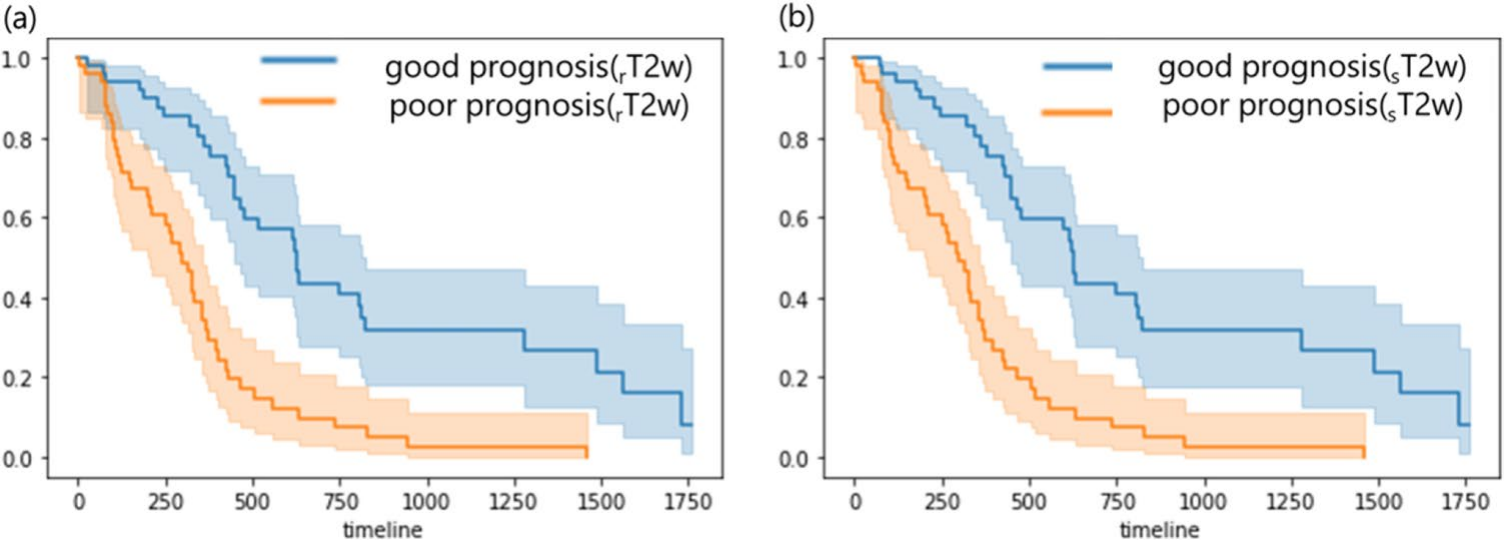
The comparison of survival curves between the good prognosis group and poor prognosis group using (**a**) rT2w-rad-score and (**b**) sT2w-rad-score, respectively

Figure 11 shows the survival curve estimated from rT2w-rad-score and sT2w-rad-score. In the two groups estimated by the rT2w-rad-score, the survival time of the good prognosis group was longer than that of the poor prognosis group (*p* < 0.05). Similarly, in the two groups estimated by the sT2w-rad-score, the survival time of the good prognosis group was longer than that of the poor prognosis group (*p* < 0.05).

**Fig. 12:**
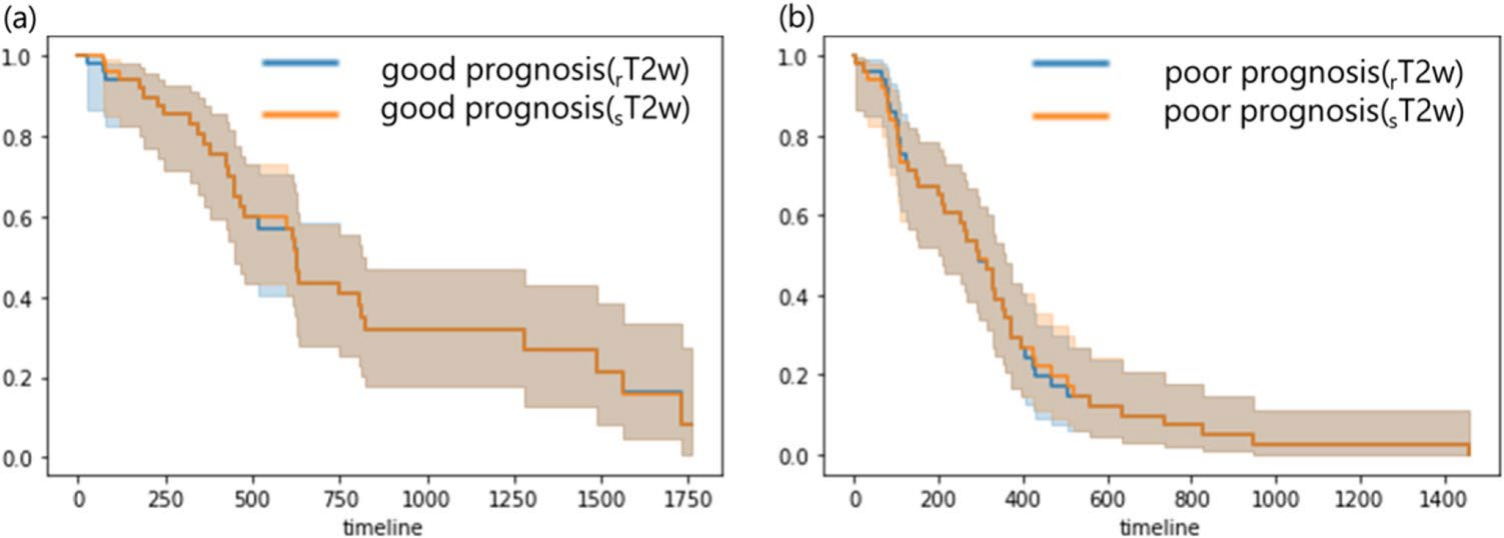
The comparison of survival curves of the rT2w-rad-score and sT2w-rad-score in (**a**) good prognosis and (**b**) poor prognosis

Figure 12 shows a comparison between the two groups estimated from the rT2w-rad-score and sT2w-rad-score for each survival curve. There was no significant difference in survival curves in the good prognosis group estimated from rT2w-rad-score and sT2w-rad-score, respectively (*p* = 0.99). Similarly, there was no significant difference in survival curves in the poor prognosis group estimated from rT2w-rad-score and sT2w-rad-score, respectively (*p* = 0.98).

**Fig. 13:**
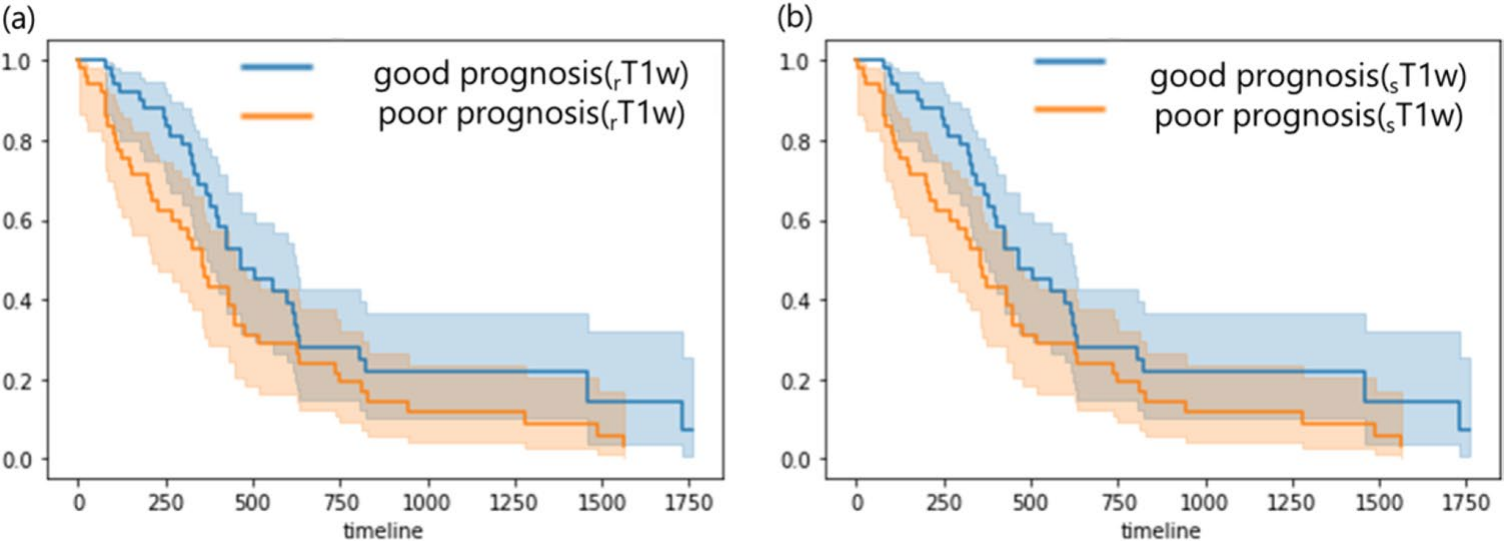
The comparison of survival curves in the good prognosis group and poor prognosis group using rT1w-rad-score (**a**) and sT1w-rad-score (**b**), respectively

Figure 13 shows the survival curves estimated from the rT1w-rad-score and sT1w-rad-score. In the two groups estimated by the rT1w-rad-score, the survival curve in the good prognosis group was no significant difference from that in the poor prognosis group (P = 0.05). Similarly, in the two groups estimated by sT1w-rad-score, the survival curve in the good prognosis group had no significant difference from that in the poor prognosis group (P = 0.05).

**Fig. 14:**
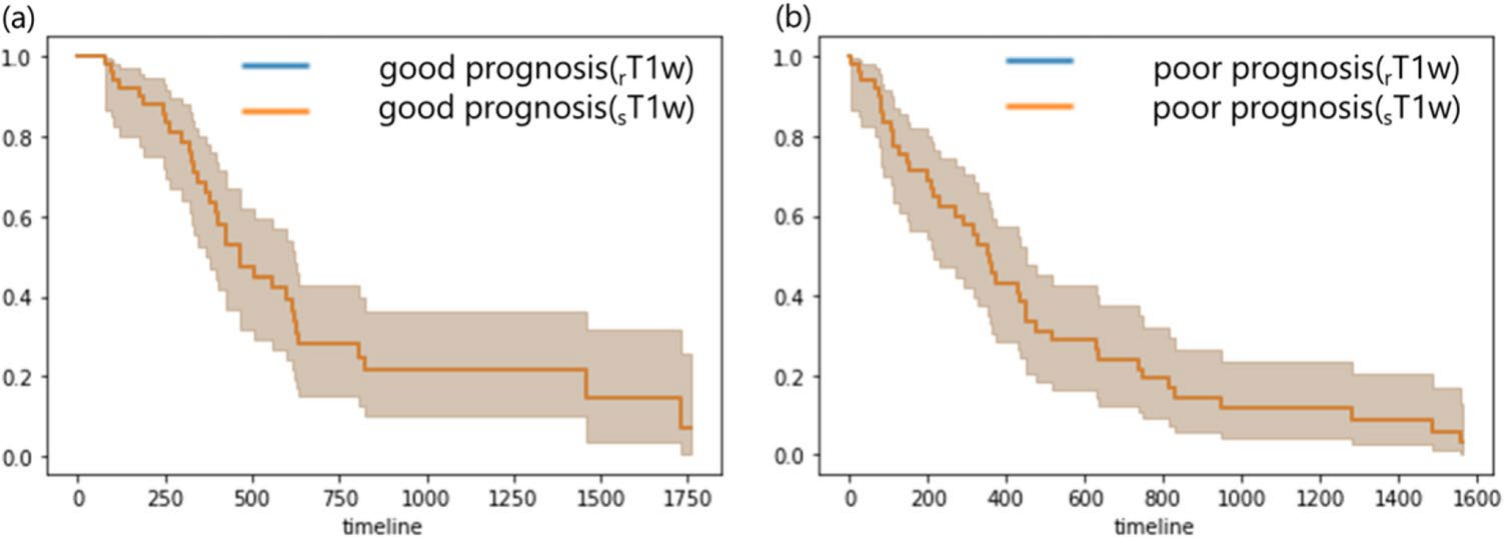
The comparison of survival curves of the rT1w-rad-score and sT1w-rad-score in (**a**) good prognosis and (**b**) poor prognosis

Figure 14 shows a comparison between the two groups estimated from the rT1w-rad-score and sT1w-rad-score for each survival curve. There was no significant difference in survival curves in the good prognosis group estimated from rT1w-rad-score and sT1w-rad-score, respectively (p = 1.00). Similarly, there was no significant difference in survival curves in the poor prognosis group estimated from rT1w-rad-score and sT1w-rad-score, respectively (p = 1.00).

## Discussion

In this paper, different contrast MRI image was on contrast MRI images for T1w or T2w using CycleGAN. Yang et al. proposed to synthesize brain MRI using conditional GAN [64]. Yang et al. proposed a cross-modality MRI style transfer framework using a generator with both an L1 loss term and an adversarial discriminator conditioned. The PSNR, MI, and SSIM between rT2w and sT2w were 22.56, 0.862, and 0.866, respectively, which were lower than the current study. The PSNR, MI, and SSIM between sT1w and rT1w were 22.52, 0.777, and 0.854, respectively, which were also lower than the current study. In another previous study, Wang et al. proposed a 3D cross-modality MRI image network [39]. Although our proposed method is trained in 2D, the values of PSNR and SSIM were higher in the current study. These results suggest that CycleGAN can synthesize brain MRI with high accuracy. However, it is unclear whether it can be used in clinical if only the image quality evaluation. Several studies on synthesized images for the dose calculation have been reported [65, 66]. Few papers report whether synthesized images can be used for prognosis prediction [29]. The current study evaluated whether style transfer can be used for prognosis prediction. For the imaging features of rT1w and sT1w, 2 common features were obtained by LASSO-Cox regression. These features were converted to rad-score for prognosis prediction. There was no significant difference in the good prognosis group estimated by rT1w-rad-score and sT1w-rad-score, respectively. There was no significant difference in the poor prognosis group as well. Hence, it is suggested that the synthesized images were equivalent to the real images even when compared by imaging features. For rT2w and sT2w, 18 common features were obtained by LASSO-Cox regression. The survival curves estimated from rT2w-rad-scores and sT2w-rad-scores have a significant difference between the good prognosis group and the poor prognosis group. Zhang et al. showed that the imaging features extracted from T2w correlated with Ki-67 expression were useful for determining prognosis [67]. Among the imaging features common to rT2 and sT2 that we extracted, log-sigma-2–0-mm-3D_glrlm_GrayLevelNonUniformity, wavelet-HLL_gldm_GrayLevelNonUniformity, and log-sigma-5–0-mm-3D_gldm_DependenceNonUniformity were ranked from those with the highest contribution in the construction of rad-score. These imaging features are involved in Gray level variation, homogeneity, etc. In the study by Pak et al. gldm_SmallDependenceEmphasis_NE_Vegldm_SmallDependenceHighGrayLevelEmpha sis_NE_Ve, firstorder_Energy_NE_Ve and other features have been extracted [24]. Their methods are not directly comparable due to differences in the way statistics are taken and the images used. However, the extracted features are consistent with our study in that they represent the complexity of the texture. In Addition, the tumor information in the T2w was reported to be related to tumor cellularity [68, 69]. The imaging features in the T2w have a possibility to show a predictor of the OS related to gene expression and tumor cellularity. There was no significant difference in the good prognosis group estimated by rT2w-rad-score and sT2w-rad-score, respectively. There was no significant difference in the poor prognosis group as well. Hence, it is suggested that the composite images are comparable to the real images when compared in terms of image features.

Multi-contrast image datasets are known to be useful in the diagnosis of GBM [70–72]. In addition, such data sets may show some missing image data [29]. Style transfer can also help to supplement such data. Thus, prognosis prediction using synthetic images is useful, although challenges remain. It was also suggested that synthesizing “prognostic level images” could be utilized as one of the landing points in the category of style transfer.

Our study has several limitations. The imaging features extracted from T1w and T2w were used for GBM prognosis prediction. Yan et al. showed that attributes obtained from CE-T1w and FLAIR are particularly useful for GBM subtyping [73]. GBM MRI images show different features of the tumor, such as heterogeneity patterns in enhanced tumor (ET) regions, non-enhanced tumor (NET) regions, and edema around the tumor (ED), which may influence prognostic prediction [69]. The sample size of datasets containing multi-contrast MRI images is limited. However, multi-contrast MRI has been found to be more accurate in predicting prognosis [74]. We aimed to improve the accuracy of prognostic prediction by complementing the missing modalities. In the future, we will expand the variety of multi-modality images through image synthesis and increase the segmentation for radiomics analysis from synthesized images such as ED in the FLAIR.

Moreover, tumor resection status and specific image features have been suggested to significantly influence predictive power, and Lasocki et al. have demonstrated that certain image features, such as FLAIR mismatch and enhancement [75] and other features such as necrosis and calcification, indicate specific glioma genotypes. In the further study, we will incorporate clinical factors into the prediction model. Furthermore, the architecture in the image synthesis affects the quality of the synthesized image. Recently, an attention mechanism has been used in the image synthesis. Since it has been shown that the Attention mechanism improves the accuracy of GAN synthetization [76], we expect to improve the accuracy by introducing the Attention mechanism to CycleGAN.

## Conclusion

The proposed method using CycleGAN was able to synthesize images with the same accuracy as the real images. Moreover, our proposed CycleGAN-based prognosis model is comparable to the conventional prognosis model with real images. It could reduce the cost and time for the image scan, which leads to promoting building the patients’ outcome prediction with multi-contrast images.

## Figures and Tables

**Fig. 15:**
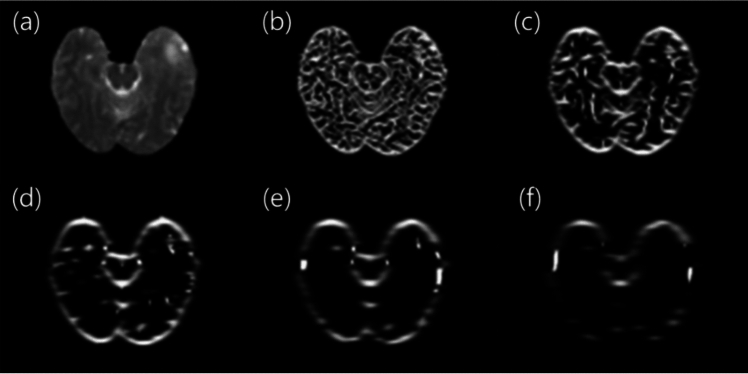
An example of image processing by LoG filter (**a**) original; (**b**) $\sigma$ = 1 mm; (**c**) $\sigma$ = 2 mm; (**d**) $\sigma$ = 3 mm; (**e**) $\sigma$ = 4 mm; and (**f**) $\sigma$ = 5 mm. $\sigma$ is standard deviation of pixel values

**Fig. 16:**
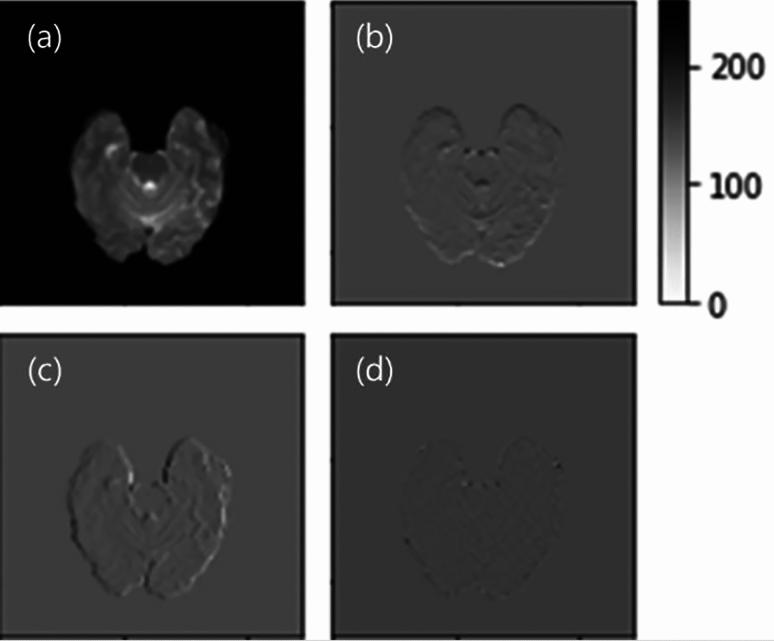
An example of image processing by 2D-wavelet transform (**a**) Approximation; (**b**) horizontal detail; (**c**) vertical detail; (**d**) diagonal detail. The color bar in the figure was set by the maximum value of pixel

## Definition of radiomics features

Shape features describe the shape of the traced region of interest (ROI) and its geometric properties such as volume, maximum diameter along different orthogonal directions, maximum surface, tumor compactness, and sphericity. [30]

First-order statistics features describe the distribution of individual voxel values without concern for spatial relationships. These are histogram-based properties reporting the mean, maximum, uniformity, etc. [30]

Second-order features include the so-called textural features [77], obtained by calculating the statistical inter-relationships between neighboring voxels [77]. They provide a measure of the spatial arrangement of the voxel intensities and intra-lesion heterogeneity [35]. The second-order features we have extracted are as follows:

## GLCM

The GLCM is a matrix defined on an image, which is a particular interest in the field of medical image analysis. The GLCM extracts the features of (Autocorrelation, Joint Average, Cluster Prominence, etc.) [55].

## GLSZM

The GLSZM quantifies gray level zones in an image. A gray level zone is defined as the number of connected voxels that share the same gray level intensity. The GLSZM extracts the features of (Small Area Emphasis, Large Area Emphasis, Gray Level Non-Uniformity, etc.) [55].

## GLRLM

The GLRLM method is a way of extracting higher-order statistical texture features based on a gray level run. The gray level run can be described as a line of pixels in a certain direction with the same intensity value. The number of times such a pixel appears is called the run length value. The GLRLM extracts the features of (Short Run Emphasis, Long Run Emphasis, Gray Level Non-Uniformity, etc.) [55].

## GLDM

The GLDM quantifies gray level dependencies in an image. A gray level dependency is defined as the number of connected voxels within distance δ that are dependent on the center voxel. GLDM extracts these features of small dependence emphasis, large dependence emphasis, gray level non-uniformity, etc. [55].

More details on the second-order features can be found in the official PyRadiomics documentation [55].

Higher-order features are obtained by filtering the image. These features are used to identify repeating and non-repeating patterns, suppress noise, and obtain detail enhancement. These include LoG filter, wavelet transform, etc., which can extract areas with increasingly coarse texture patterns [30]. These filters are defined as follows.

## LoG filter

The LoG filter is known as a filter that can highlight tumor characteristics not visible in the original image [77]. The LoG filter is difined as

A1 $$\text{LoG}\left(x, y\right)=-\frac{1}{\pi {\sigma }^{4}}\left[1-\frac{{x}^{2}+{y}^{2}}{2{\sigma }^{2}}\right]{e}^{-\frac{{x}^{2}+{y}^{2}}{2{\sigma }^{2}}}$$

where the LoG filter is the second-order derivative of the Gaussian filter. The Gaussian filter is difined as

A2 $$g\left(x,y\right)=\frac{1}{2\pi \sigma }{e}^{-\frac{{x}^{2}+{y}^{2}}{2{\sigma }^{2}}}$$

where partial differentiation about $x$,

A3 $$\frac{\partial }{\partial x}g\left(x,y\right)=\frac{-x}{2\pi {\sigma }^{4}}{e}^{-\frac{{x}^{2}+{y}^{2}}{2{\sigma }^{2}}}$$

where further partial differentiation about $x$,

A4 $$\frac{{\partial }^{2}}{\partial {x}^{2}}g\left(x,y\right)=\frac{{x}^{2}-{\sigma }^{2}}{2\pi {\sigma }^{6}} {e}^{-\frac{{x}^{2}+{y}^{2}}{2{\sigma }^{2}}}$$

where the LoG filter is defined as the addition of the second-order partial derivative for $x$ and the second-order partial derivative for $y$.

$$\frac{{\partial }^{2}}{\partial {x}^{2}}g\left(x,y\right)+\frac{{\partial }^{2}}{\partial {y}^{2}}g\left(x,y\right)=\frac{{x}^{2}+{y}^{2}-2{\sigma }^{2}}{2\pi {\sigma }^{6}} {e}^{-\frac{{x}^{2}+{y}^{2}}{2{\sigma }^{2}}}$$

A5 $$=-\frac{1}{\pi {\sigma }^{4}}\left[\frac{-\left({x}^{2}+{y}^{2}\right)+2{\sigma }^{2}}{2{\sigma }^{2}}\right]{e}^{-\frac{{x}^{2}+{y}^{2}}{2{\sigma }^{2}}}$$

$$=-\frac{1}{\pi {\sigma }^{4}}\left[1-\frac{{x}^{2}+{y}^{2}}{2{\sigma }^{2}}\right]{e}^{-\frac{{x}^{2}+{y}^{2}}{2{\sigma }^{2}}}$$

In addition, the LoG filter changes the kernel with parameters called $\sigma$ [mm] and filter size.

For example, when these parameters are $\sigma$ = 3 mm and filter size = $5\times 5$, then kernel K is as

A6 $$\text{K}=\left[\begin{array}{ccccc}0& 0& 1& 0& 0\\ 0& 1& 2& 1& 0\\ 1& 2& -16& 2& 1\\ 0& 1& 2& 1& 0\\ 0& 0& 1& 0& 0\end{array}\right]$$

The parameters of $\sigma$ [2.0 to 5.0] were used. Figure 15 shows an example of image processing by the LoG filter. The LoG filter can produce different textures depending on the value of $\sigma$ [mm].

Wavelet transform.

The wavelet transform is known as a method to obtain multiscale textures [78]. The wavelet transform uses the discrete wavelet transform (DWT). The wavelet transform is defined as

A7 $$\left({W}_{\psi }f\right)\left(b, a\right)=\frac{1}{\sqrt{a}}\bullet {\int }_{-\infty }^{\infty }\overline{{\psi \left( {\frac{{x - b}}{a}} \right)}}\bullet f\left(x\right)dx$$

where a is scale, b is translation, and $\psi \left(x\right)$ is called the mother wavelet.

The original function can be obtained as

A8 $$f\left(x\right)={C}_{\psi }^{-1}{\int }_{-\infty }^{\infty }da{\int }_{-\infty }^{\infty }db\,{a}^{-2}\left({W}_{\psi }f\right)\left(b, a\right)\,\psi \left(\frac{x-b}{a}\right)$$

DWT is a discretization of the wavelet transform with $a$ and $b$ as $a={2}^{-j}$ and $b={2}^{-j}k$, respectively. DWT is defined as

A9 $${d}_{k}^{\left(j\right)}={2}^\frac{j}{2}\bullet {\int }_{-\infty }^{\infty } \overline{{\psi \left( {2^j x - k} \right)}} \bullet f\left(x\right)dx$$

In addition, we used the 3D-DWT, which is represented by the following tensor product.${V}^{3}=\left({L}^{x}\oplus {H}^{x}\right)\otimes \left({L}^{y}\oplus {H}^{y}\right)\otimes \left({L}^{z}\oplus {H}^{z}\right)$

A10 $$={L}^{x}{L}^{y}{L}^{z}\oplus {L}^{x}{H}^{y}{L}^{z}\oplus {H}^{x}{L}^{y}{L}^{z}\oplus {H}^{x}{H}^{y}{L}^{z}$$

$$  \oplus L^{x} L^{y} H^{z}  \oplus L^{x} H^{y} H^{z}  \oplus H^{x} L^{y} H^{z}  \oplus H^{x} H^{y} H^{z}  $$,

Where $\oplus$ and $\oplus$ denote a space direct sum and convolution, respectively, *H* and *L* denote high-pass and low-pass filters; $x$, $y$, and $z$ are the 3D coordinates. Consequently, we extracted these features of multiscale texture types (LLL, HLL, LHL, HHL, LLH, HLH, LHH, and HHH). Mostly, the fine texture is derived from the details (i.e., high-pass filter), while the coarse texture is derived from the approximation (i.e., low-pass filter) [79].

Figure 16 shows an example of image processing by 2D-wavelet transform. In this example, there are four textures that can be obtained for 2D: LL, LH, HL, and HH. These correspond to image approximations, horizontal detail, vertical detail, and diagonal detail. The Fig. 16 shows the rT2w by wavelet transform.
